# Blue rings in trees and shrubs as indicators of early and late summer cooling events at the northern treeline

**DOI:** 10.3389/fpls.2024.1487099

**Published:** 2025-01-22

**Authors:** Agata Buchwal, Pawel Matulewski, Ylva Sjöberg, Alma Piermattei, Alan Crivellaro, Angela Balzano, Maks Merela, Luka Krže, Katarina Čufar, Alexander V. Kirdyanov, Tatiana Bebchuk, Tito Arosio, Ulf Büntgen

**Affiliations:** ^1^ Institute of Geoecology and Geoinformation, Faculty of Geographical and Geological Sciences, Adam Mickiewicz University, Poznan, Poland; ^2^ Department of Geography, University of Cambridge, Cambridge, United Kingdom; ^3^ Department of Ecology and Environmental Science, Umeå University, Umeå, Sweden; ^4^ Department of Agricultural, Forest and Food Sciences, Università degli Studi di Torino, Torino, Italy; ^5^ Forest Biometrics Laboratory, Faculty of Forestry, “Stefan cel Mare” University of Suceava, Suceava, Romania; ^6^ Department of Wood Science and Technology, Biotechnical Faculty, University of Ljubljana, Ljubljana, Slovenia; ^7^ Sukachev Institute of Forest SB RAS, Krasnoyarsk, Russia; ^8^ Global Change Research Institute CAS, Brno, Czechia; ^9^ Department of Geography, Faculty of Science Masaryk University, Brno, Czechia

**Keywords:** blue rings, treeline, Fennoscandia, *Pinus sylvestris*, *Juniperus* spp. shrubs, cooling events, cell wall lignification

## Abstract

The high temperature sensitivity of pine trees in northern Fennoscandia has led to some of the most reliable tree-ring climate reconstructions in the world for the past millennia. However, wood anatomical anomalies that likely reflect temperature-induced reductions in cell wall lignification, the so-called Blue Rings (BRs), have not yet been systematically investigated in trees and shrubs in northern Europe. Here, we present frontier research on the occurrence of BRs in *Pinus sylvestris* trees and *Juniperus communis* (L) s.l. shrubs from the upper treeline in northern Norway (69°N) in relation to instrumental temperature data covering the last ca. 150 years. The highest number of BRs was found in 1902, with 96% of *Pinus* trees and 68% of *Juniperus* shrubs showing BRs. These corresponded on average to a 42% vs. 27% proportion of the growth ring in 1902 which was less-lignified in *Pinus* trees and *Juniperus* shrubs, respectively. Another peak in BRs recorded for 1877 was more pronounced in *Pinus* trees (88%) than in *Juniperus* shrubs (36%), with a lower proportion of less lignified rings. We found the lowest monthly sums of growing degree days in June 1902 and August 1877, resulting in more uniform non-lignified BRs in 1902 than in 1877. Prolonged early growing season cooling shortened the growing season in 1902 and resulted in much thinner cell walls in trees and shrubs than in 1877, which was characterized by extended cooling at the end of the growing season. Also, after 1902 BR, *Pinus* trees exclusively showed no recovery in the mean cell wall thickness in the following year. Our study provides the first evidence for different impacts of early versus late growing season cooling on cell wall lignification in trees and shrubs at the northern treeline. Using the anatomy of BRs, we demonstrated the potential to refine summer cooling event reconstructions at an intra-annual resolution in northern Fennoscandia and beyond.

## 1 Introduction

Although most recent studies focus on climate warming, tracing records of past cooling events can provide valuable insights into climate extreme variability and the growth responses of woody plants to these extremes. Many previous studies based on tree rings performed cold climate reconstructions using frost ring analyses at the treeline habitats, including studies conducted on conifer trees (Brunstein, 1996; Gurskaya, 2021; LaMarche and Hirschboeck, 1984; Panayotov et al., 2013; Payette et al., 2010; Tardif et al., 2020) and shrubs (Hantemirov et al., 2000, 2004). Northern Fennoscandia is one of the most studied regions in terms of tree rings and climate (Linderholm et al., 2010). Based on dendroclimatological studies of *Pinus sylvestris*, the region provides summer temperature reconstructions that span up to 7,400 years (Grudd et al., 2002). Many of these reconstructions represent summer temperature signals for the Northern Hemisphere (Briffa et al., 1988; Büntgen et al., 2011; Esper et al., 2012; Grudd, 2008). However, the strongest temperature signal for the Fennoscandia region is not reconstructed from the tree ring widths, but from the cell wall characteristics of *Pinus sylvestris*, based, for example, on maximum latewood density (Briffa et al., 1988; Büntgen et al., 2011; Grudd, 2008). Moreover, the latest development of quantitative wood anatomical (QWA) studies increased climate fidelity and obtained a climatic signal that cannot be acquired from ring-width data alone. For example, a recently established 1,170-year-long chronology based on anatomical measurements of tracheids of *Pinus sylvestris* provided critical insights into climate dynamics in the Fennoscandia region and assessed the modern period to be warmer than the medieval climate (Björklund et al., 2023). Systematic application of QWA thus has the potential to provide a broader understanding of past climate and refine climate reconstructions performed on highly temperature-sensitive woody species at the northern treeline. Northern treeline is a habitat not only for trees, but also for shrubs, such as conifer *Juniperus* spp., that are highly sensitive to temperature variabilities (Hallinger et al., 2010; Hantemirov et al., 2011). This shrub species is known to be long-lived (Carrer et al., 2023; Lehejček et al., 2024), with individuals up to 840-years old (Hantemirov et al., 2004), and therefore along with the trees could be used for long-term paleoclimate reconstructions based on wood anatomical properties.

Recently, the use of a double-staining technique on thin wood sections – with a mixture of Safranin and Astra Blue dye – has allowed researchers to gain insights into the effects of cold temperature on the lignification process. Blue Rings (BRs) (Crivellaro et al., 2018; Piermattei et al., 2015) are a relatively newly described anatomical feature in conifers with axial tracheid cell walls stained blue due to a lack of lignin, usually stained in red (Gärtner and Schweingruber, 2013). In the current literature, the formation of BRs is associated with cold growing season conditions in different species of the genus *Pinus*, such as *Pinus nigra* Arnold at the alpine treeline in the central Apennines (Piermattei et al., 2015), *Pinus contorta* in NW North America (Montwé et al., 2018), *Pinus uncinata* Mill. in the central Pyrenees (Piermattei et al., 2020), *Pinus longaeva* D.K. Bailey in Nevada, USA (Tardif et al., 2020) and *Pinus sylvestris* L. in Romania (Semeniuc et al., 2016), Latvia (Matisons et al., 2020), Poland (Matulewski et al., 2022), Estonia (Greaves et al., 2023), and northern Finland (Björklund et al., 2021; Büntgen et al., 2022). In contrast to trees, the potential of BRs in shrubs for paleoclimate studies still has to be explored. Studies on the formation of BRs in the cold-resistant woody plants at the treelines can potentially verify thermal thresholds for cell wall lignification in plants growing at the margin of their distribution, and thus for woody plants in general.

The number of studies on species-specific BRs has gradually increased in recent years. However, the main research gaps related to the formation and quantification of BRs still need to be addressed: i) Is BR formation species-specific, and how coherent is it across different woody plant forms at a specific geographical location? ii) How much do the QWA characteristics change in BRs, and are they specific to cooling events? To address these questions, we focused on the elevational treeline in northern Scandinavia where Scots pine and *Junipers* spp. coexist and potentially show BRs in their annual growth rings. We selected the study site considering the long-term instrumental data of daily air temperature recorded since 1877. We aimed to understand the temperature-related lack of lignification and thus to explore if specific QWA characteristics of the BRs are related to intra-seasonal temperature variations during the ca. 150-years long instrumental period.

## 2 Materials and methods

### 2.1 Study site

**Figure 1:**
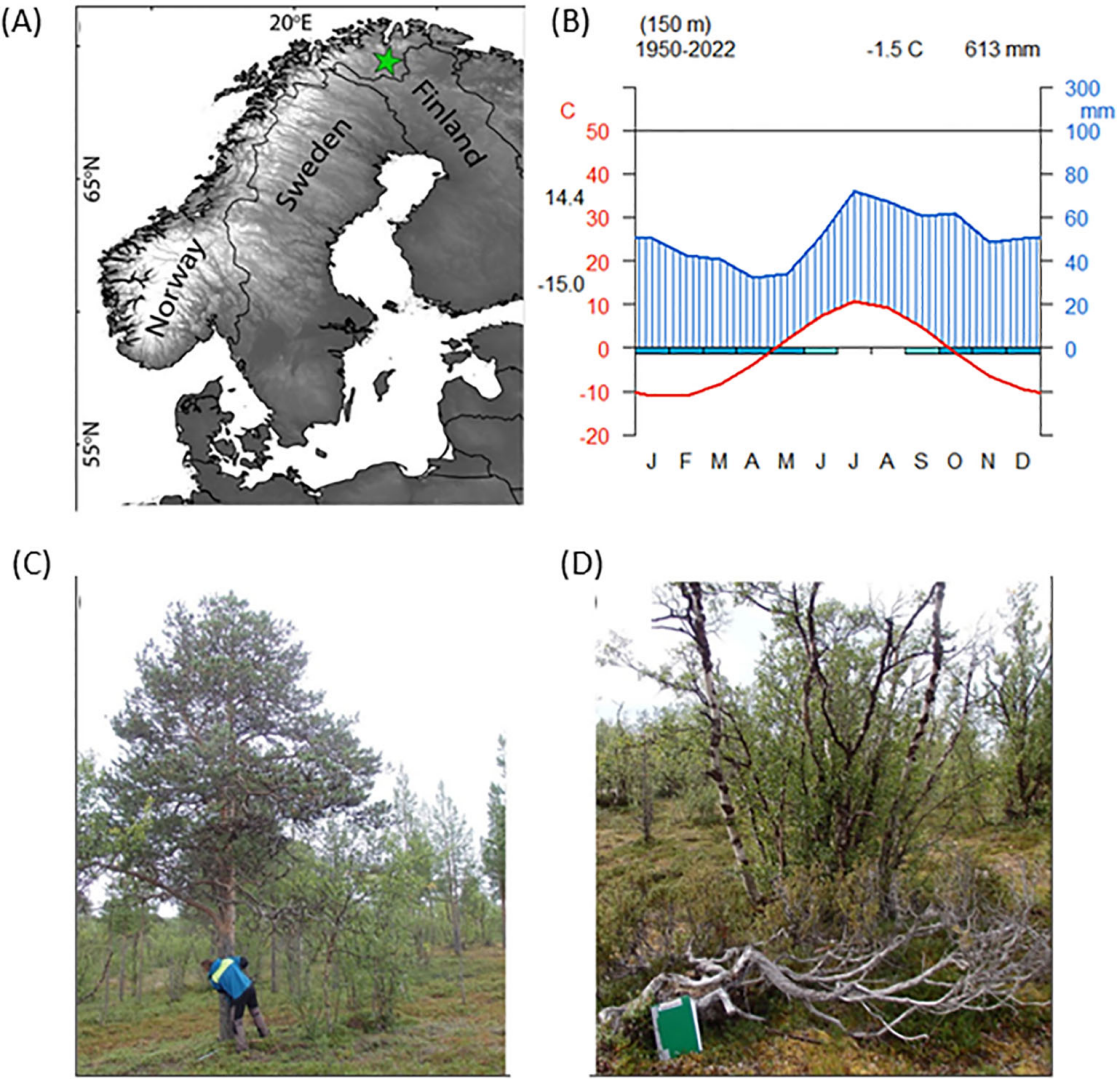
**(A)** Study site location (green star) in northern Norway; **(B)** climatic diagram for Karasjok area (data source: E-OBS 29.0e, period 1950-2022); **(C)** overview of an upper treeline habitat and examples of a *Pinus* tree and **(D)** a *Juniperus* shrub that were sampled.

The study site was located on Mount Iškoras in NE Norway (69°N, 25°E), at the northern treeline (**Figure 1**). To study woody plants at the cold limits of their growth (Körner, 2012) we sampled shrubs from the shrubline and upper treeline (300-450 m asl), and trees from the upper treeline (280 m asl). The mean annual temperature, as measured down in the valley in Karasjok (ca. 150 m asl), is -1.7°C (period 1877-2022), with July (13.3°C, standard deviation (sd) = 1.9°C) and January (-15.2°C, sd = 4.3°C) being the warmest and coldest months, respectively. The annual precipitation is 370 mm (period 1877-2022), with the monthly maximum in July (64 mm, sd = 36 mm). The respective climatic diagram for the period 1950-2022 is presented on **Figure 1B**. The diagram was plotted using homogenized meteorological data (E-OBS 29.0e; Cornes et al., 2018) using climatol package (Guijarro, 2006) in R.

### 2.2 Study species

In total, we sampled 25 *Pinus sylvestris* (thereafter *Pinus*) trees and 54 *Juniperus communis* (L.) *s.l.* (thereafter *Juniperus*) shrubs. We sampled two cores at breast height (i.e., 1.3 m) of living trees, from the upslope and the downward side of a tree, and we hand-sawed at least one stem-base disc from both dead (n = 8) and living shrubs. The main above-ground shoot was selected from multi-stem shrubs, and to improve the cross-dating the selected *Juniperus* shrubs were subjected to serial sectioning (Kolishchuk, 1990). We selected 25 cross-dated (i.e., correlated at *r* > 0.15 with the master chronology) *Juniperus* shrubs for a comparative analysis with 25 *Pinus* trees.

### 2.3 Thin-sectioning and growth ring width measurements

Thin cross-sections of 15-30 microns thickness were obtained from *Pinus* tree cores and *Juniperus* discs using a sledge GSL microtome. A double-staining procedure was performed using a mixture of Safranin and Astra Blue dye (Gärtner and Schweingruber, 2013), where 1g Safranin and 0.5 g of Astra blue were dissolved in 100 ml of distilled water and mixed in the ratio of 1:2. The staining procedure took 3-5 minutes and excess dye was removed by ethanol baths of increasing concentration and finally by absolute ethanol. The sections were permanently mounted on microscope slides using Canada balsam and oven-dried for at least 24 h at 60°C (Gärtner and Schweingruber, 2013).

Multiple single images were taken using an Olympus BX43 microscope (Tokyo, Japan) and Olympus CS30 digital camera at x200 magnification. The single digital images were stitched using the PTGui software (http://www.ptgui.com) to produce a panorama image of the disc and cores’ cross-sectional cut. Ring width measurements for both species were performed on digital panorama images of an entire shrub disc or a tree core, using WinCELL (Regent Instruments, Canada) and ImageJ Fiji software (Schindelin et al., 2012), respectively. *Juniperus* shrubs manifested very irregular growth patterns, with most rings wedging and the pith often missing in the old samples. Therefore, growth rings for *Juniperus* shrubs were measured along 4-5 radii within a single disc. Cross-dating of both shrubs and trees was verified using COFECHA software (Grissino-Mayer, 2001; Holmes, 1983). Detrending of raw series for trees and shrubs was performed with 2/3 spline (dplR, Bunn et al., 2021) using each individual full timespan, extending beyond the instrumental period. Residual chronologies (i.e., autocorrelation removed) covering the instrumental period were used in the subsequent climate-growth relationship analyses. Descriptive statistics were computed for both chronologies (**Table 1**), including: mean sensitivity (MS) quantifying the year-to-year variability; the Gini coefficient (Biondi and Qeadan, 2008) and interseries correlation (r) as a synthetic measures of inter-series heterogeneity among detrended chronologies; the expressed population signal (EPS) that measures the reliability of the chronology based on interseries correlations and sample size (Wigley et al., 1984); subsample signal strength (SSS; (Buras, 2017; Cook and Kairiukstis, 1990); first-order auto-correlation (AC (1)) expressing the influence of growth of the previous year on the current year growth.

**Table 1:** Descriptive statistics for standardized *Pinus sylvestris* trees and *Juniperus* spp. shrubs chronologies (common period: 1877-2022) from Mount Iškoras, NE Norway.

species	# sample	MS	Gini	AR(1)	r	snr	EPS	SSS
*Pinus sylvestris*	25	0.24	0.19	0.57	0.41	17.01	0.944	0.998
*Juniperus spp.*	25	0.31	0.23	0.55	0.14	3.67	0.786	0.947

MS, mean sensitivity; Gini, Gini coefficient; AR(1), first-order autocorrelation; r, mean interseries correlation; snr, signal-to-noise ratio; EPS, expressed population signal; SSS, subsample signal strength.

### 2.4 Wood anatomy

The frequency of the occurrence of BRs and Frost Ring (FRs) was counted for both species for the instrumental period (1877-2022) using a light microscope at x200 magnification. Following the protocol proposed by (Piermattei et al., 2020), we categorized two types of BRs: i) a complete BR as a continuous blue zone of axial tracheids with often both inner and outer cell walls being less lignified and thus showing the lowest rate of cell wall lignification; (ii) a partial BR as a discontinues blue zone (or solitary blue tracheids) with mainly inner cell walls being less lignified. The QWA measurements were conducted on the most frequently formed BRs, i.e., for the calendar years in which at least five trees and five shrubs showed BRs. Specifically, QWA analyses were performed on two BR years, plus one ring preceding and one following the blue ring year. Two cell parameters, such as tangential cell wall thickness (CWT) and cell lumen radial diameter (CL) (Cuny et al., 2019), were measured manually using a segment line tool in the ImageJ Fiji software (Schindelin et al., 2012) along three radial rows of tracheids (randomly selected from the upper, middle and lower portions of the image). Additionally, we calculated the proportion of the less lignified growth ring and expressed it as the percentage of the ring width being less lignified. In contrast to BRs, FRs were characterised by distorted xylem tissue (callus tissue) damaged by freezing in the growing season during which the cells of the tissue were formed (Fritts, 1976).

### 2.5 Climate-growth relationship analyses

Daily temperature data from the Karasjok meteorological station (located ca. 20 km away from the study site at 129 m asl) were acquired for the period 1877-2022 (https://seklima.met.no/; station ID SN97251 and SN97250). The daily records were not gap-free (**Supplementary Figure 1**) and the gap years were excluded; however, the comparison with temperature records from the other stations showed that most likely the gap years were not extremely cold (**Supplementary Figure 2**). Growing degree days were calculated as the sum of temperatures for all summer days (i.e., from June to August) with a daily temperature >5°C. Since instrumental data from Karasjok station were not-gap free, we performed bootstrapped correlation analyses (period 1901-2022) using CRU TS 4.07 temperature and precipitation data (Harris et al., 2023).

## 3 Results

### 3.1 Tree and shrub-ring chronologies

**Figure 2:**
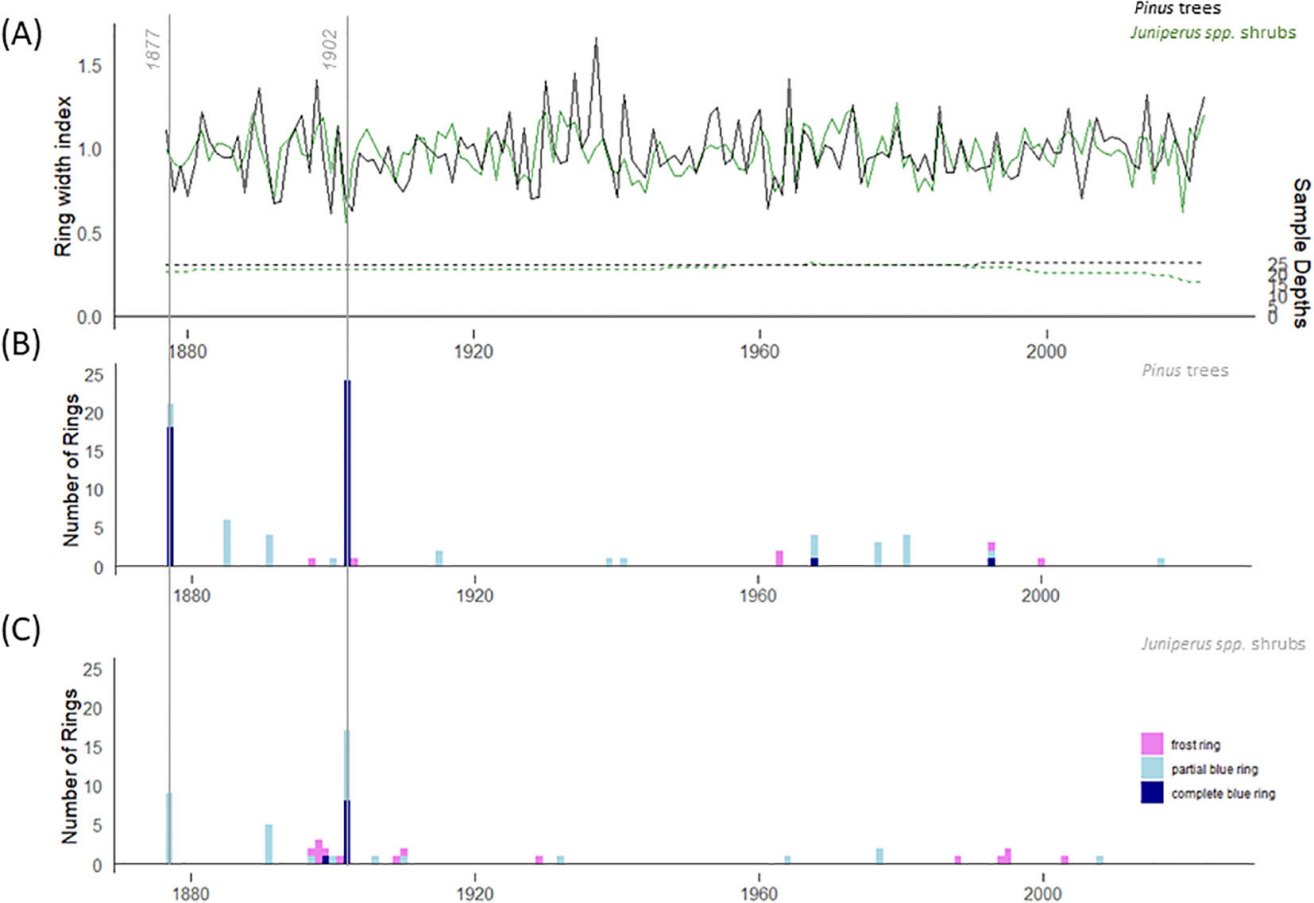
**(A)** Residual tree-ring chronologies for *Pinus sylvestris* trees (black) and *Juniperus* spp. shrubs (green) for the instrumental period (1877-2022); Frequency of complete and partial blue rings and frost rings in **(B)** *Pinus trees* and **(C)** *Juniperus* spp. shrubs.

The instrumental temperature period (1877-2022) was covered with 24 and 21 *Pinus* trees and *Juniperus* shrubs, respectively (**Figure 2A**). The mean tree age was 268 years, and the mean ring width was 0.551 mm (min = 0.021, max = 2.915). The mean shrub age was 282 years and the mean ring width was 0.073 mm (min = 0.005, max = 0.839). In total, 15 missing rings were detected at the single shrub level, and no missing rings were detected at the single tree level. Descriptive statistics, including interseries correlation, were higher for the *Pinus* chronology (r = 0.41) than for the *Juniperus* (r = 0.14) chronology (**Table 1**).

### 3.2 Climate sensitivity

**Figure 3:**
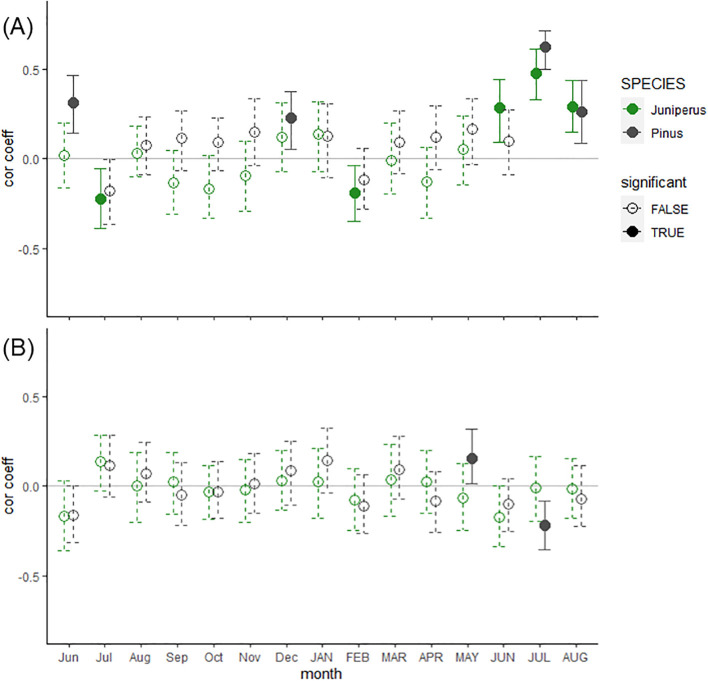
Bootstrapped correlation coefficients between residual *Pinus sylvestris* trees (black) and *Juniperus* spp. shrubs (green) chronologies from Mount Iškoras (NE Norway) and **(A)** air temperature and **(B)** precipitation CRU TS 4.07 data for the common period (1901-2022). Significant coefficients (P < 0.05) together with associated confidence intervals (at the level of 95%) are marked with solid lines and filled dots. Monthly climatic variables include the period from the previous year’s June until the current August (capital letters).

Both species were sensitive to mean July temperature (period 1901-2022), with a higher positive correlation coefficient for the *Pinus* chronology (r = 0.62; confidence intervals (CI) =0.51:0.72) than for the *Juniperus* (r = 0.48, CI = 0.32:0.61) chronology. The *Juniperus* chronology was also positively correlated with mean temperature in June (r = 0.28, CI = 0.10:0.44) and August (r = 0.29, CI = 0.14:0.43), and the *Pinus* chronology was also correlated to August temperature (r = 0.27, CI = 0.08:0.45) (**Figure 3A**). The climate growth responses for *Pinus* have also shown a positive correlation with the temperatures for the previous June (r = 0.32, CI = 0.13:0.47) and December (r = 0.23, CI = 0.08:0.38). The *Juniperus* shrub chronology was insensitive to precipitation, while the *Pinus* tree chronology indicated a positive correlation with May precipitation and a negative correlation with July precipitation (**Figure 3B**).

### 3.3 Blue rings characteristics

For the 146-year long instrumental period (1877-2022), the total number of BRs was higher for *Pinus* trees (n = 74) than for *Juniperus* shrubs (n = 41) (**Figures 2B, C**). Overall, 2.1% rings (74 out of 3,536 total rings measured) in the *Pinus* trees and 1.3% growth rings (41 out of 3,224) in the *Juniperus* shrubs were BRs. The highest occurrence of BRs was found in the year 1902, with 96% (n = 24) of Pinus trees and 68% (n = 17) of *Juniperus* shrubs forming BRs. The second highest occurrence of BRs, during the instrumental period, was found in 1877, with 84% (n = 21) of *Pinus* trees and 36% (n = 9) of *Juniperus* shrubs showing BRs. A high frequency of BRs (i.e., a minimum of five BRs) was also found in the trees in 1885 (n = 6/25 trees) and in the shrubs in 1891 (n = 5/25 shrubs).

**Figure 4:**
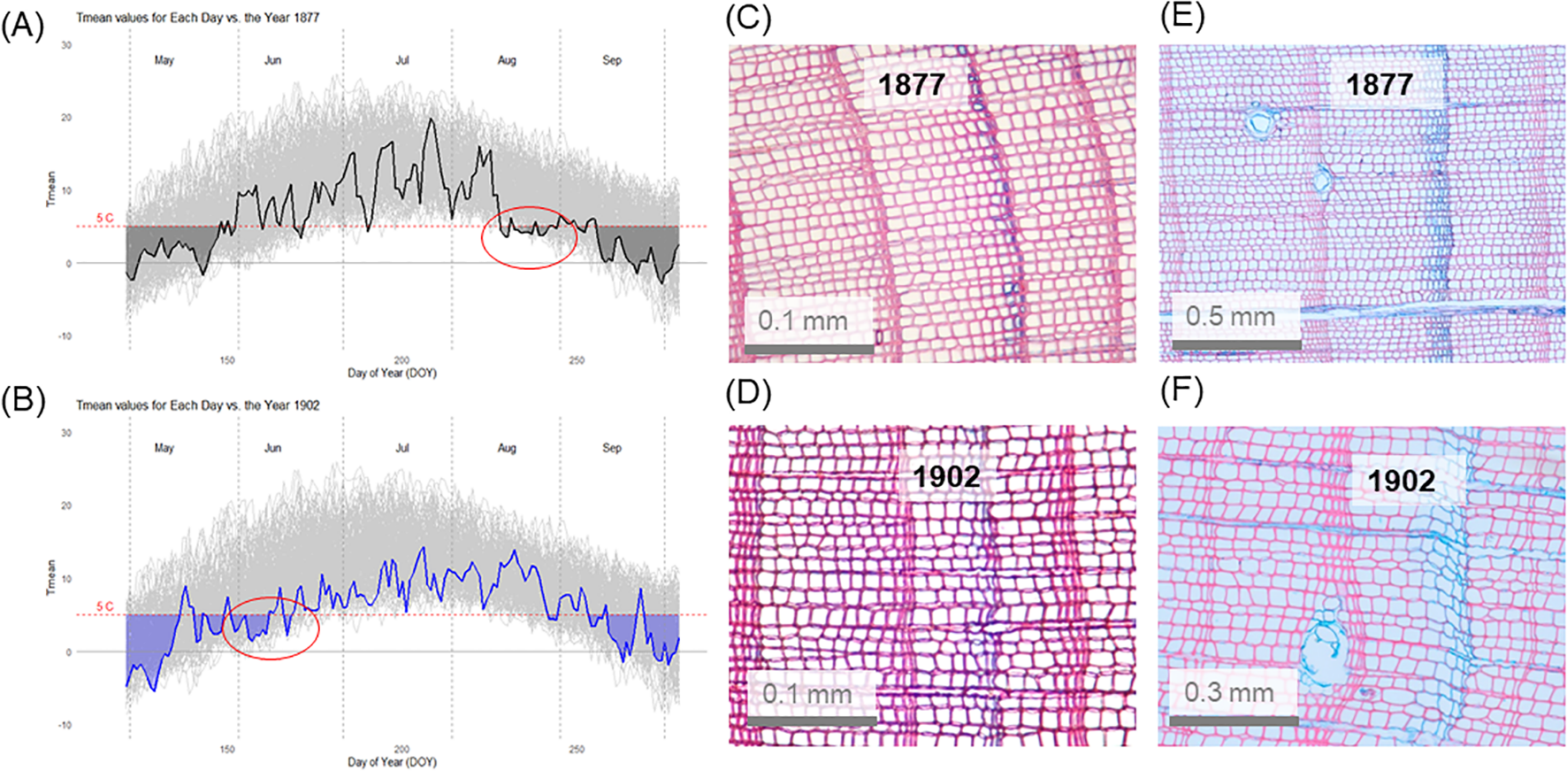
Daily temperature data for the instrumental period (1877-2022, grey lines) with temperature conditions shown for the blue ring years, **(A)** 1877 and **(B)** 1902. Days with a daily temperature < 5°C are shaded in grey for 1877 and in blue for 1902. Example of blue rings in Juniperus spp. shrubs **(C, D)** and Pinus sylvestris trees **(E, F)** found in 1877 and 1902. Note the highest proportion of a ring being less lignified in year 1902 in pine **(F)**. A partial blue ring is shown in **(C)**, and complete blue rings are shown in **(D–F)**.

In all BRs, the less lignified cells were mainly located at the end of the growth ring, i.e., in the latewood or at the transition between early and latewood. We did not find any less lignified cells in the first rows of earlywood cells, neither in *Pinus* trees nor in *Juniperus* shrubs. A total of 59.5% of BRs in the *Pinus* trees (**Figures 4E, F**) and 22% in the *Juniperus* shrubs (**Figure 4D**) represented complete BRs. The *Pinus* trees formed in total 40.5% of partial BRs, whereas *Juniperus* shrubs formed 78% of such BRs (**Figure 4C**). In addition to BRs, frost rings (FRs) were also formed in *Juniperus* shrubs (n = 14; 0.4% of the total rings measured), but in *Pinus* trees we encountered only 6 FRs (0.2%). In contrast to BRs, the FRs were randomly distributed across the study period.

### 3.4 Quantitative wood anatomy of blue rings

**Figure 5:**
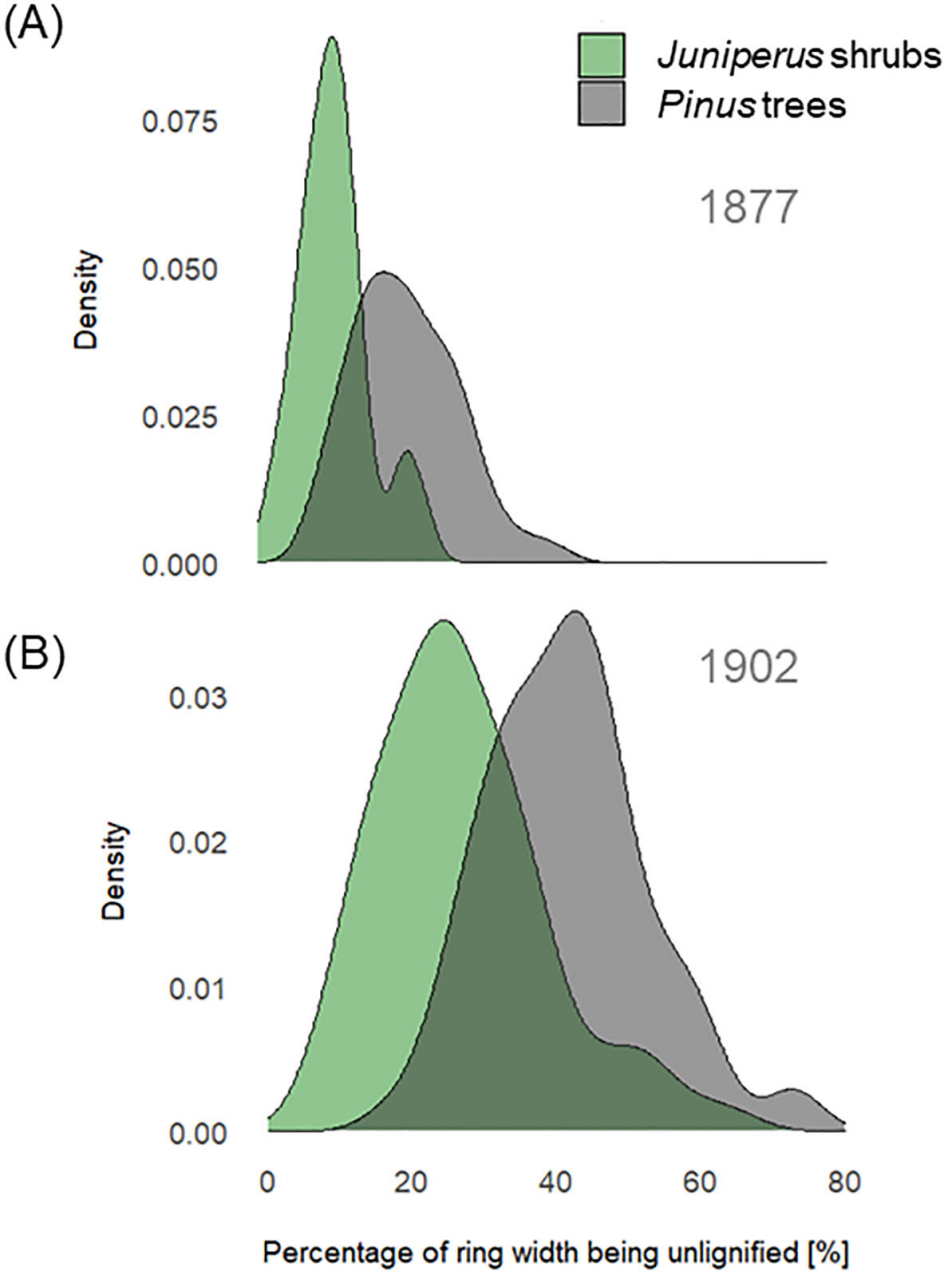
Percentage of ring width being less lignified in year 1877 **(A)** and 1902 **(B)** *for Juniperus* spp. shrubs (green) and *Pinus sylvestris* trees (grey). Density distributions show the variation in unlignified ring width percentages across samples for each species group.

The mean percentage of the ring width being less lignified in 1877 was 27% (min = 8%, max = 63%) in the *Pinus* trees and 11% (min = 2%, max = 23%) in the *Juniperus* shrubs (**Figure 5A**). In contrast, in 1902, the mean percentage of the ring width being less lignified was higher in both cases, at 42% (min = 17%, max = 73%) for the *Pinus* trees and 27% (min = 8%, max = 62%) for the *Juniperus* shrubs (**Figure 5B**). These results correspond to six (min = 2, max = 15) and two (min = 1, max = 4) less lignified cells formed on average in 1877 in a single radial file, and to seven (min = 3, max = 15) and three (min = 1, max = 6) less lignified cells formed in 1902, in the *Pinus* trees and the *Juniperus* shrubs, respectively. The number of less lignified cells was highly correlated with the ring width in the *Pinus* trees (yr 1902: *r* = 0.90, *p* < 0.001, df = 68, vs. yr 1877 *r* = 0.65, *p* < 0.001, df = 64), but for the *Juniperus* shrubs, this was only true in 1902 (yr 1902: *r* = 0.76, *p* < 0.001, df = 46, vs. yr 1877 *r* = 0.28, *p* = 0.278, df = 22). The numbers of less lignified cells for 1902 and 1877 in the *Pinus* trees were also significantly correlated with the total number of cells per radial file (yr 1902: *r* = 0.61, *p* < 0.001, df = 69; yr 1877: *r* = 0.58, *p* < 0.001, df = 64). Whereas, for the *Juniperus* shrubs, this relationship was significant for 1902 (*r* = 0.56, *p* = 0.001, df = 46), but not for 1877 (*r* = 0.16, *p* = 0.449, df = 22).

**Figure 6:**
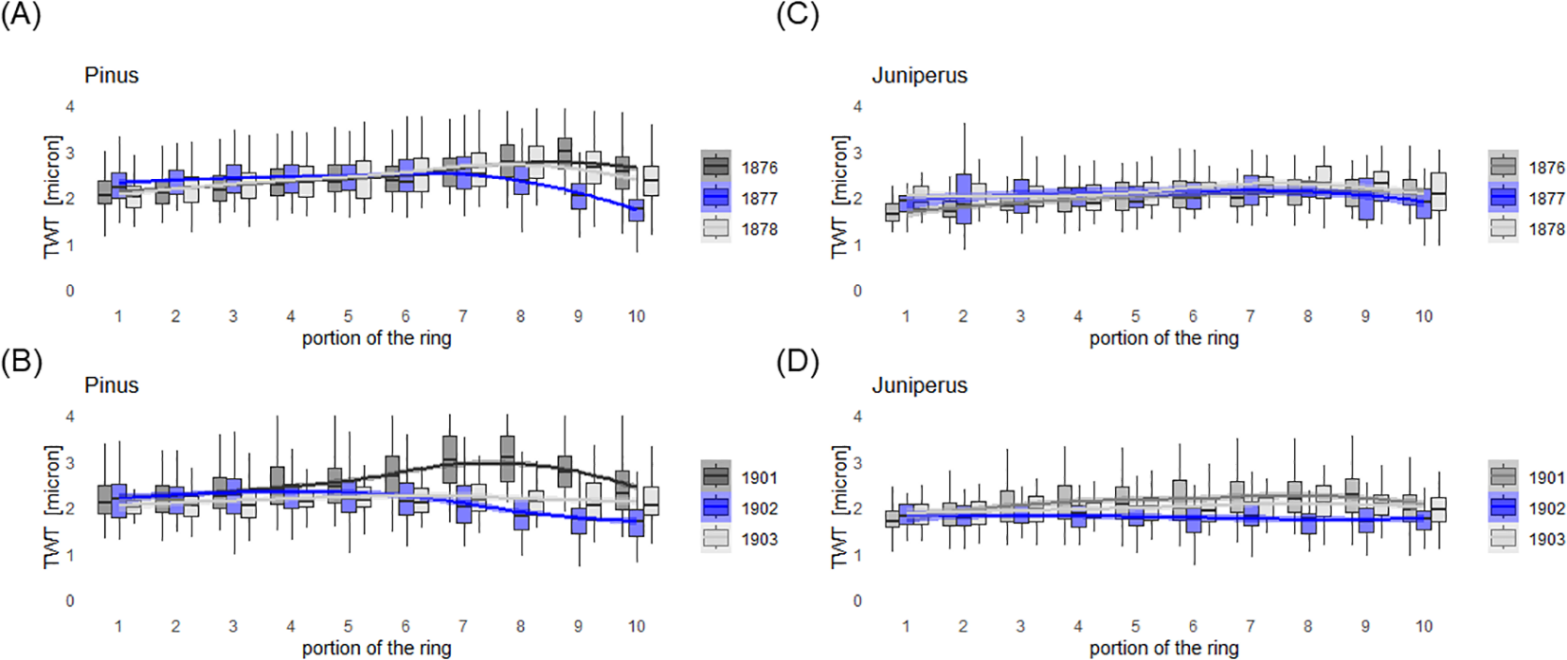
Changes in tangential cell wall thickness (TWT) for the blue ring formed **(A, C)** in 1877 and **(B, D)** in 1902 for *Pinus* trees **(A, B)** and *Juniperus* shrubs **(C, D)**. For comparison, measurements of TWT in a ring proceeding (dark grey) and following (light grey) the respective blue ring are shown. A portion of a ring is defined from 1 (i.e., earlywood) to 10 (i.e., latewood).

The mean CWT values for less lignified cells in 1877 were 2.0 (min = 1.3, max = 2.9) microns in the *Pinus* trees and 1.9 (min = 1.2, max = 2.6) microns in the *Juniperus* shrubs. In 1902, the mean CWT values decreased to 1.8 (min = 1.1, max = 2.9) microns for the *Pinus* trees and 1.7 (min = 0.91, max = 2.4) microns for the *Juniperus* shrubs. The differences in mean CWT of the less lignified cells in the BRs between 1902 and 1877 were statistically significant for both the *Pinus* trees (*P* < 0.001) and *Juniperus* shrubs (*P* < 0.01). The mean diameter of the radial cell lumen (CL) of less lignified cells was significantly greater for the BRs in 1902 compared to 1877 in the *Pinus* trees (19.2 *vs.* 14.9 microns) and *Juniperus* shrubs (6.2 *vs.* 4.2 microns). Additionally, we observed no recovery in CWT in the year 1903, i.e., following the most distinctive BR that formed in 1902, but only in the *Pinus* trees (**Figure 6B**). Such an anatomical response of the wood was not observed in 1877, which was characterized by late summer cooling, neither in the *Pinus* trees (**Figure 6A**) nor in the *Juniperus* shrubs (**Figure 6C**).

### 3.5 Blue rings and climate

Climate data revealed that the year 1902 was characterized by the lowest mean monthly temperature in June (5.3°C), and August 1877 was the second coldest (7.6°C) after the year 1899 (7.5°C) in the period 1877-2022. Specifically, June 1902 was 4.6°C colder than the average June (mean June temperature = 9.9°C, sd = 2.0°C), and it was the coldest month during all growing seasons in the period 1877-2022. August 1877 was 3.3°C colder than the average for August in the same period.

Daily temperature data (despite some gaps; **Supplementary Figure 1**) revealed that June 1902 and August 1877 were characterized by the lowest sums of growing degree days (GDD) with 123.1 (June 1902) and 182.2 (August 1877) GDD. This corresponds to exactly 12 days characterized by a mean daily temperature <5°C both in June 1902 and August 1877. The longest period with consecutive days colder than 5°C in August 1877 lasted five days (August 20-25) (**Figure 4B**), and ten days between June 1-10 in 1902 (**Figure 4A**). If we consider the BRs formed in other years, then in both 1885 and 1891 cold periods in the first part of June were observed (**Supplementary Figure 3**). Unfortunately, the climate data for 1885 are not complete, which limits the analytical power of this investigation.

## 4 Discussion

### 4.1 Blue rings in trees *versus* shrubs

Our comparative study showed that despite being of different life forms and botanical genera, conifer woody plants from the same geographical locations form less lignified growth rings in response to extreme cold conditions in the same years. The *Pinus* trees were more prone to the formation of BRs than the *Juniperus* shrubs. The *Pinus* trees recorded the extreme early summer cooling event in such way that almost half of the growth ring width was less lignified in 1902 versus just a small part of the ring being less lignified in 1877. In addition, and in the *Pinus* trees alone, there was no recovery in the mean CWT after the 1902 BR, i.e., in 1903, which was not among the coldest years (**Supplementary Figure 5**). This could be due to the short and generally cold growing season in 1902, which was thus not long and warm enough for the production of the sufficient amount of carbon reserves and so they were less available for cell wall thickening in the following year. In fact, the mean CWT in 1903 was as small as in the 1902 BR, which suggests that after the extreme cooling event (here, in the early summer of 1902), there is a carry-over effect in a reduced rate of carbon assimilation and deposition in the both early and latewood tracheids (**Figure 6**).

*Juniperus* shrubs seem to be more adapted to extreme summer cooling, and especially to late growing season cooling since the occurrence of BRs in 1877 in the shrubs compared to trees was ca. 2.5 times lower. Similar findings were reported about light ring formation in the Polar Ural Mountains, with light rings being very rare in the wood of Siberian juniper, in contrast to larch trees (Hantemirov et al., 2004). Light rings represent latewood zone with thin-walled cells, usually in trees at northern and subalpine timberlines (Filion et al., 1986). But in contrast to BRs light rings do not inform about lack of lignification in the cell walls, since light rings are assessed on a polished wood surface and not on a double-stained thin-section. Due to the much narrower latewood expressed in a lower number of latewood tracheids (Liang et al., 2012), *Juniperus* shrubs appear to be less prone to form BRs due to growing season cooling events. Moreover, ring formation in the *Juniperus* shrubs seems to start and cease earlier than in the *Pinus* trees. For example, earlier completion of wood formation and thus a shorter growing season was found in *Pinus mugo* shrubs in comparison to *Picea abies* trees at the alpine treeline in Central Europe (Treml et al., 2019). suggested that trees with wide cells at the stem base require more time for cell differentiation and maturation than shrubs with narrow cells. Indeed, we observed a more significant proportion of a growth ring being formed and lignified in the *Juniperus* shrubs in comparison to the *Pinus* trees at the time of material sampling on August 11 (**Supplementary Figure 4**). The earlier onset of the growing season can potentially be supported in the *Juniperus* shrubs’ growth ring widths being positively correlated with June temperatures. In contrast, such a relationship for the *Pinus* trees was insignificant (**Figure 3A**). On the other hand, a positive correlation with early season temperatures might expose the *Juniperus* shrubs to higher FRs formation than *Pinus* trees. The higher replication of BRs found in the *Pinus* trees compared to *Juniperus* shrubs, combined with distinct anatomical features (i.e., narrower CWT), predispose them to serve as a proxy for intra-seasonal summer cooling events at the northern treeline.

### 4.2 Early and late summer cooling events recorded in blue ring anatomy

Summer temperature conditions in 1902 and 1877 were particularly unfavourable for the growth of woody plants. In June 1902, the temperatures were above 5°C, which might be considered as a thermal threshold for cambial activity (Rossi et al., 2007), only starting from June 18. And according to long-term data (1877-2022), the daily temperatures above 5°C are usually present in the study area from the last week of May (May 29) and in mid-June (i.e., from June 16) the mean daily temperatures usually exceed 10°C. In 1902, due to low early June temperatures, the onset of the growing season was most probably delayed until the second or even the third week of June, which is about three weeks later than usual. Considering that annual ring formation in *Pinus sylvestris* lasts for about seven weeks in the northern boreal forest (Schmitt et al., 2004; Seo et al., 2011), such a delay accounted for almost half of the expected time of cambial activity. Consequently, we observed that the coldest June recorded in 1902 and generally cold summer in that year (**Supplementary Figure 2**), resulted in a much greater proportion of a less lignified growth ring. Following (Siekacz et al., 2024), we claim that a cold June might define how long cambial activity can be sustained, and thus the length of the growing season. We assume that due to delayed onset of cambial production, the lignification of latewood tracheids was not yet completed when cold temperatures arrived later in the season and disrupted the process of lignification, which then lead to the formation of a BR (c.f., Siekacz et al., 2024). The effect of a cold June should thus be considered as an indirect effect of cold early season conditions on BR formation in the year 1902.

In contrast, the late summer cooling event in August 1877 was less pronounced in the anatomy of BRs. This was observed in both studied species, but a common signal was higher for the *Pinus* trees than for the *Juniperus* shrubs. Specifically, for the *Pinus* trees, the greater proportion of a growth ring width being less lignified in 1902 (i.e., 42%) than in 1877 (27%) allowed for differentiation between late and early summer cooling events. Considering that earlywood width in the *Pinus* trees as a rule amounts to about 75% of the annual ring width (as assessed for *Pinus sylvestris* site in northern Finland, Seo et al., 2011) this means that not only latewood but also some earlywood tracheids were less lignified during the summer of 1902 in the *Pinus* trees. In contrast, the *Juniperus* shrubs demonstrated stronger adaptation to summer cooling events than the *Pinus* trees. Still, a similar difference in the proportion of ring width being less lignified was observed in the shrubs, with more than twice the rate recorded in 1902 (27%) compared to 1877 (11%).

It is well known that latewood density in *Pinus* trees at the northern treeline depends on the temperature during the most active growth period, estimated to last from June 24 to August 7, beyond the continuous northern forest line in Finland (Tuovinen, 2005). Schmitt et al. (2004) claimed that the growth of the earlywood in *Pinus* trees from northern Finland does not start to form until mid-June, and the latewood formation ends in late August. Similarly, in northern Siberia, it was found that for *Larix* trees maximum latewood density is significantly correlated with the temperature over a relatively long period of the growing season, i.e., from early June to even early October (Vaganov et al., 1999). Later, Kirdyanov et al. (2003) showed that in eastern Subarctic Siberia, the latewood cell wall thickness correlates with almost the entire period with a temperature >0°C, i.e., from the end of May until the middle or end of August. They also indicated that the enlargement and wall-thickening stages of cell development require several weeks, i.e., from 1.5 to 2.5 months, in larch trees at the northern treeline. This, together with our study on the anatomy of BRs, points out that the duration of cell wall deposition and lignification depends on a much longer period than just the late part of the growing season, and a lack of lignification in a given year might be related to both early and late unfavourable growing season conditions.

Previous studies have indicated the impact of exceptionally low temperatures, specifically in the second half of the growing season, on light ring formation, i.e., growth rings characterized by particularly low-density latewood (Filion et al., 1986; Gindl, 1999). Gindl (1999) revealed that the lignin content in the secondary cell wall layer of the terminal latewood tracheids in spruce from the Alps was positively correlated with the late season temperature, i.e., from the beginning of September until the third week of October. However, it should be noted that BRs and light rings are both characterized by low values of CWT in the latewood tracheids, but they do not represent the same anatomical feature. For example, not all thin cell walls are less lignified in the light rings. Still, due to the late discovery of BRs, it is studies on light rings that provide our knowledge on the limiting factors in cell wall lignification processes. The early summer cooling most likely postponed the onset of the growing season, and thus reduced its length in 1902. As a result, the time for cambial activity was shortened together with the subsequent phases of wood cell development, including the period for latewood cell wall deposition and lignification in the *Pinus* trees. A similar pattern was observed by Gurskaya (2019) when examining light rings in various larch tree species across the forest-tundra ecotone in northern Siberia. She indicated that with a cold June, the process of cell division is retarded, resulting in the formation of a small number of latewood tracheids and a narrow latewood zone. Additionally, Gurskaya (2019) has indicated the impact of low August temperatures on light ring formation in larch trees due to delayed cell wall maturation, which could be related to our observation of the formation of BRs in both trees and shrubs in the summer of 1877.

Despite the cold and strongly delayed onset of the growing season in 1902, most earlywood cells were formed in a “regular” way (i.e., lignification of the cell walls was accomplished). Still, the season was too short for the formation and lignification of typical radially flattened thick-walled latewood cells. Montwé et al. (2018) revealed that distinctive BRs followed by FRs in *Pinus contorta* from western North America are linked to a late initiation of the growing season, an early end of the growing season, and a generally cool growing season. Meanwhile, Wang et al. (2000) indicated a cold spring as a major factor causing light ring formation in *Picea mariana* in northern Quebec. They also pointed out that a cold spring and the formation of light rings might occur due to cooling caused by a volcanic eruption, which affects the late onset of the growing season and limited time for latewood formation. Björklund et al. (2021) assumed that reduced cell wall lignification is associated with unfavourable climatic conditions because the synthesis and deposition of lignin requires extensive photo assimilation. On the other hand, the secondary wall thickening of wood cells in *Pinus sylvestris* was shown to be mainly affected by temperature (Antonova and Stasova, 1993; Wodzicki, 1971). Wodzicki (1971) was probably the first researcher to observe that the final thickness of tracheid cell walls in *Pinus sylvestris* is closely related to the duration of the maturation phase over most of the season. This relation determines the transition from the thin-walled tracheids of earlywood to thick-walled latewood tracheids. Later, Vaganov et al. (2006) showed that the leading role in determining the final cell wall thickness in larch trees is played by the duration of this process, which, as we observed in the example of the 1902 blue ring, might be substantially limited by the very cold (thus short) growing season. They reported that the size of the latewood cells at the northern treeline in Siberia correlated with temperatures of three to five pentads after snow melt and that the correlation of temperature with cell wall thickness changes when the dates of pentads are fixed to snow melt rather than the calendar (Kirdyanov et al., 2003; Vaganov et al., 2006, 1999). Based on the example of larch trees from northern Siberia, Vaganov et al. (2006) claimed that if latewood tracheids are produced by the cambial zone in mid-July, they have to spend at least two weeks to expand and then three to four weeks for cell wall deposition and lignification. Trees thus need a minimum of six weeks to complete the maturation process. Examples of BRs from 1877 and 1902 indicate that cold conditions, either at the beginning or at the end of the growing season, might disrupt this process by inducing a shorter growing season both in trees and shrubs at the northern treeline. As it is generally known that differentiation of the last formed tracheids near the cambium in conifer species can continue until late autumn (Gricar et al., 2005), future studies should aim to study both the effects of growing season length and changes in thermal conditions across the seasons on the formation of BRs.

### 4.3 Blue rings and pan-regional climatic signal

The formation of BRs in 1902 and 1877 was also documented in a recent study conducted in northern Finland (Björklund et al., 2021), which indicates that summer cooling events were present in these two calendar years at a broader spatial scale and thus were potentially triggered by extra-regional factors. Indeed, these were also the coldest two years recorded in northern Finland during the instrumental period (Björklund et al., 2021) and induced coherent formation of BRs in both northern Norway and Finland, thus potentially across the larger region in northern Fennoscandia. This high spatial correlation in the formation of BRs suggests that they can serve as diagnostic rings for cooling events at the regional scale and, combined with QWA studies, can provide new characteristics of these events at intra-annual resolution. This type of information cannot be obtained from traditional tree-ring proxies, such as ring widths or maximum latewood density (MXD), and the novelty of the anatomical studies of BRs in woody plants is that they integrate both quantitative (e.g. CWT) and qualitative (e.g. lack of lignification) measures of woody plant responses to cooling events.

Previous studies have associated both blue and light ring occurrence with global climatic events such as long-term cooling after large volcanic eruptions (Filion et al., 1986; Hantemirov et al., 2004; Piermattei et al., 2020; Tardif et al., 2020). The coldest June in the instrumental record found for our study area, in 1902, might be related to the volcanic eruption of Mount Pelée (late April/early May 1902) in the eastern Caribbean. The cold conditions of August 1877 could be related to the eruption of Cotopaxi in Ecuador (late June 1877), but to the best of our knowledge, the cooling effect of this eruption has not been reported so far in northern Fennoscandia. Therefore, similar to the study on BRs in the Pyrenees (Piermattei et al., 2020), we suppose that not all BRs can be directly linked to volcanic-driven climate change, and this lack of exclusivity in the formation of BRs should be taken into account in paleoclimate studies.

### 4.4 Thermal threshold for the formation of blue rings – limitations of the study

Our study revealed that the summers of 1877 and 1902 were exceptionally cold and unfavourable for cell wall lignification in woody plants. The highest frequency of BRs in both species and related lack of cell wall lignification was found when the mean June temperature fell below 5.3°C (as in 1902), which corresponded to 123.1 GDD in June (i.e., cumulative sum of daily temperatures above 5.0°C). The second highest number of BRs in the *Pinus* trees was found for the year 1877, when the mean August temperature reached 7.6°C (i.e., 182.2 GDD). These values can be proposed as potential thermal thresholds for the formation of BRs in conifer woody plant species at the northern treeline and, interestingly enough, are similar to the thermal thresholds indicated for light ring formation in larch trees in northern Siberia. Regardless of larch species, Gurskaya (2019) showed that the normal progression of latewood formation and late tracheid maturation is possible if mean monthly temperatures are above 6°C in June and 9.5°C in August. Below these values, the formation of light rings was observed along a vast west-to-east transect in northern Siberia. The thermal threshold indicated for BRs formation based on our study conducted in northern Norway is lower, since BRs indicate not only thin-walled latewood cells (such as those found in the light rings), but primarily the less lignified cell walls.

Despite being the first study to show the potential of the anatomy of BRs for recognizing intra-seasonal cooling events, we must acknowledge the limitations of this work. In the period covered by the instrumental daily temperature data, there were only two calendar years with high BR frequencies in both the focal trees and shrubs. As such, a more thorough assessment of the thermal thresholds for the formation of BRs in the instrumental period was hampered. Nevertheless, in the light of detailed anatomical investigations performed for the BRs from 1902 and 1877, we can assume that in the *Pinus* trees, a higher ratio of the growth ring being less lignified (i.e., ca 40% of a ring width) and significantly thinner cell-walls within the BR and the following ring indicate an early growing season cooling event (or events). In contrast, late cooling events are manifested in a much more lignified BR and fully lignified tracheid cell walls in the following ring. Recognition of similar events would require anatomical measurements of BRs formed at a high frequency in both trees and shrubs in calendar years beyond the instrumental period, and this one direction for a follow-up study.

We also need to keep in mind that *in situ* temperatures, i.e., at the treeline sampling locations, were most likely lower than those measured at the meteorological station and used to quantify the initial thermal thresholds for the formation of BRs in our study. The differences in elevation between the meteorological station and sampling locations were between 170 m (for the treeline) and 300 m (for the shrubline). As such, the absolute thermal threshold values for the formation of BRs might be up to 1-2°C and up to 3°C lower for the *Pinus* trees (treeline) and *Juniperus* shrubs (shrubline), respectively. Hence, to refine the precision of using the anatomy of BRs for paleoclimate reconstructions, future studies should aim to obtain *in situ* temperature measurements and use them to assess the thermal thresholds for cell wall lignification in trees and shrubs more accurately (Gricar et al., 2005; Treml et al., 2019). It would also be insightful to verify if plant age and tree/shrub height are related to the formation of BRs. For example, Lenz et al. (2013) indicated the strong aerodynamic coupling of trees to air temperature, and thus their exposure to colder temperatures, whereas small-stature plants (such as shrubs) experience warmer tissue temperatures (Grace and Norton, 1990), and so are able to grow at higher elevations and potentially are less prone to the formation of BRs. Additionally, since the secondary wall thickening of wood cells in *Pinus sylvestris* is mainly affected by temperature (Antonova and Stasova, 1993; Wodzicki, 1971), we primarily associate the formation of BRs with low temperatures. However, future studies should also verify if the occurrence of BRs is not driven or co-driven by a lack of moisture or other non-climatic factors. Additionally, other factors that can potentially explain the variability in BRs frequency between the species, such as differences in air *versus* soil surface temperatures, snow cover duration and depths, vegetation cover density and light availability – which are all microsite dependent – should be included in future studies.

## 5 Conclusion

Our study found that at the northern treeline, BRs can be highly synchronized between conifer tree and shrub species in years characterized by the coldest summer conditions in the instrumental period. The study is also the first one to indicate that i) both early- and late-summer cooling events are related to the formation of BRs in two different growth forms of woody plants at the northern treeline, and ii) the timing of the summer cooling events is revealed in different ratios of cell walls with a lack of lignification, and different proportions of a growth ring being less lignified. Of the two species studied, *Pinus* trees appear to be less adapted to cold conditions and form a higher proportion of less lignified cells than the *Juniperus* shrubs. Therefore, BRs in *Pinus* trees might be more applicable in paleoclimate studies where the anatomy of BRs can be used as a novel proxy of past summer cooling events.

## Data Availability

The data presented in the study are deposited in the ITRDB repository, accession number https://www.ncei.noaa.gov/access/paleo-search/study/39999 (*Pinus sylvestris*) and https://www.ncei.noaa.gov/access/paleo-search/study/40000 (*Juniperus spp.*).
