# Supplementary material for: Blue rings in trees and shrubs as indicators of early and late summer cooling events at the northern treeline
Source: Front Plant Sci. 2025 Jan 22;15:1487099. doi: 10.3389/fpls.2024.1487099 (PMC11793999; doi:10.3389/fpls.2024.1487099)
Supplement: Supplementary file 1 [file DataSheet1.pdf]

Supplementary Figures

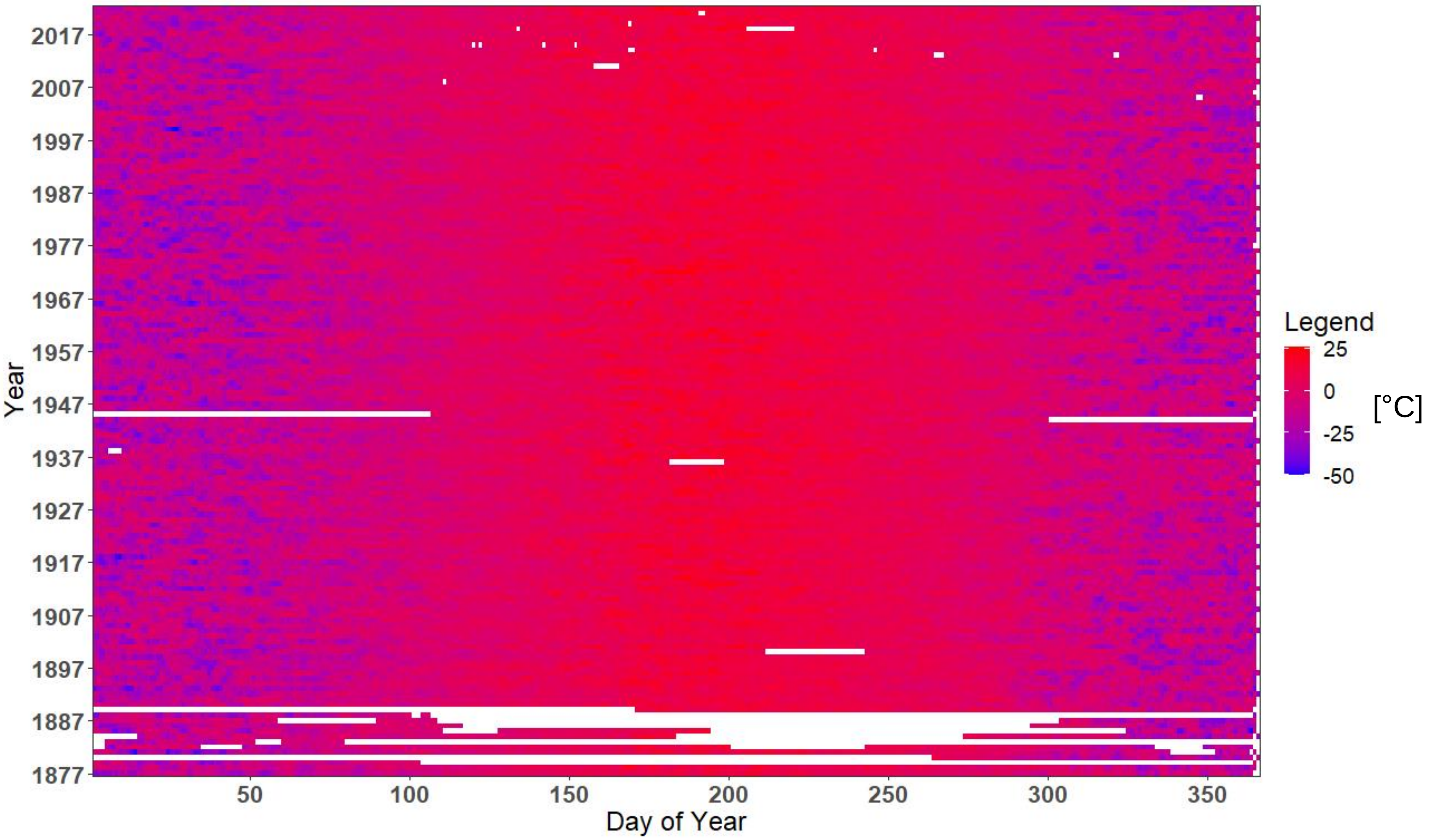

SUPPLEMENTARY FIGURE 1. Overview of daily temperature data (1877-2022) for Karasjok meteorological station (NE Norway) with data gaps marked in white. Data source: https://seklima.met.no/

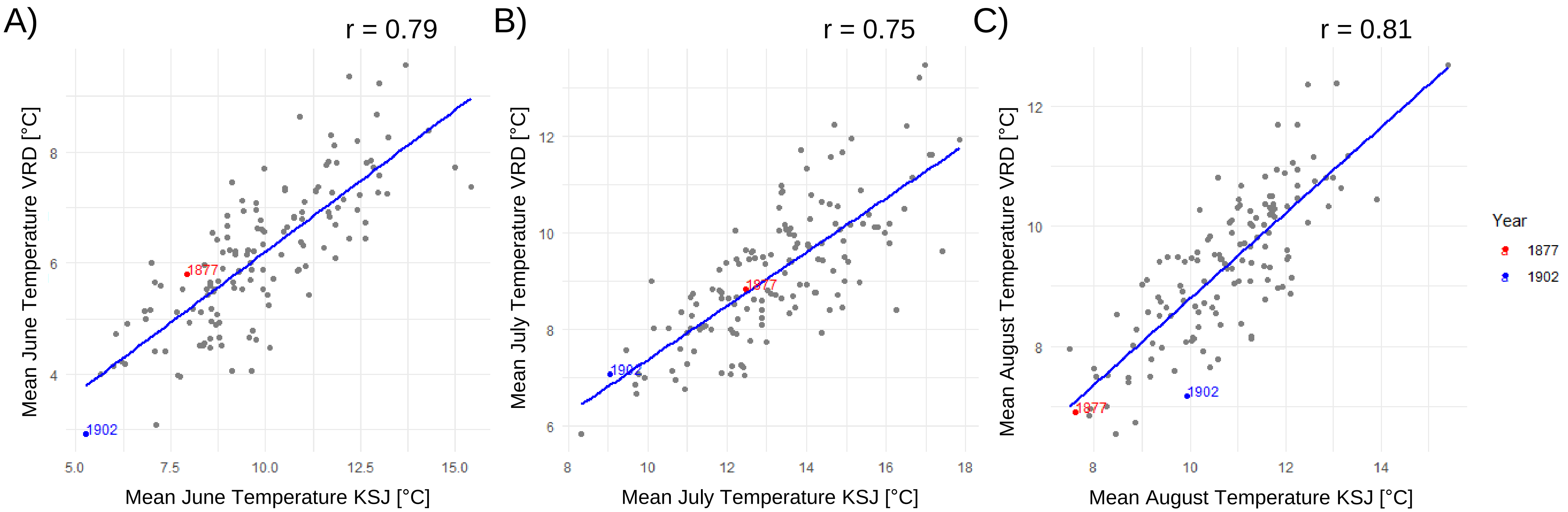

SUPPLEMENTARY FIGURE 2. Comparison between mean monthly temperature data (1877-2022) for Karasjok (KSJ) and Vardø (VRD) meteorological stations located ca. 235 km NE from Karasjok. Temperature conditions for blue ring years are marked in red and blue for year 1877 and 1902, respectively. Linear regression coefficients (r) are shown in topleft corners. Data source: https://seklima.met.no/

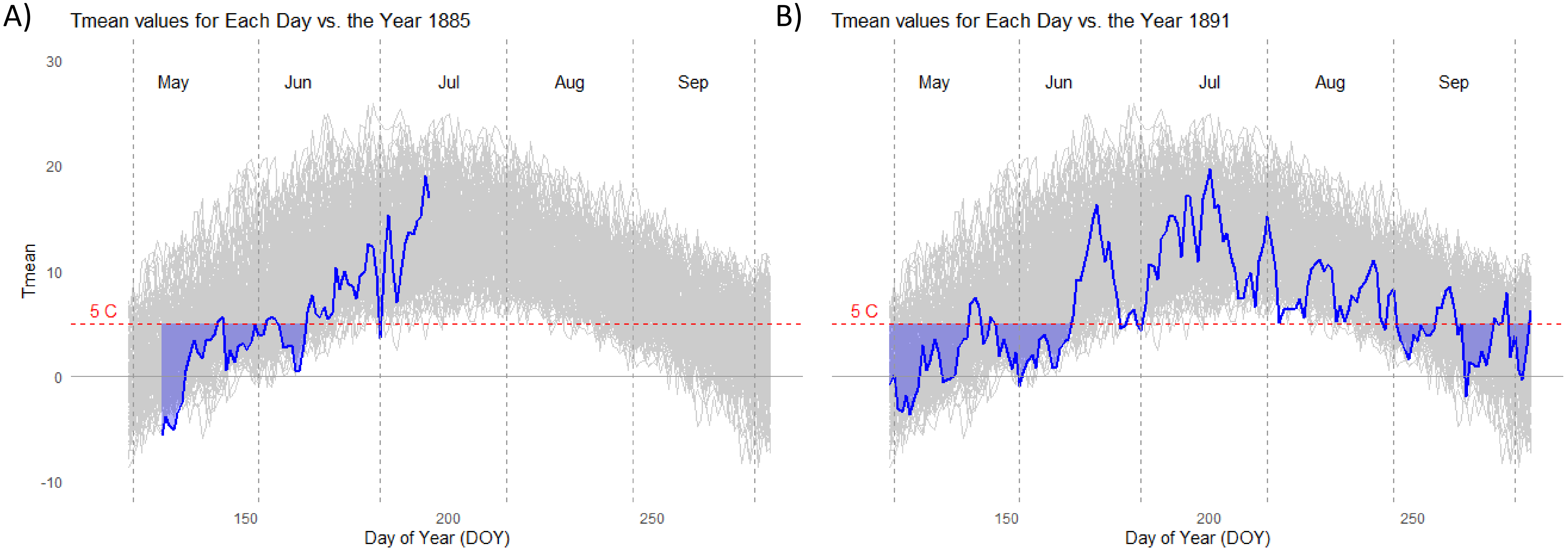

SUPPLEMENTARY FIGURE 3. Daily temperature data for the instrumental period (1877-2022, grey lines) with temperature conditions shown for blue ring years formed (A) in 1885 (n BRs = 6/25 trees) and (B) 1891 (n BRs = 5/25 shrubs). Days with daily temperature < 5°C are shaded in blue. Note data gap from mid-July in year 1885. Data source: https://seklima.met.no/

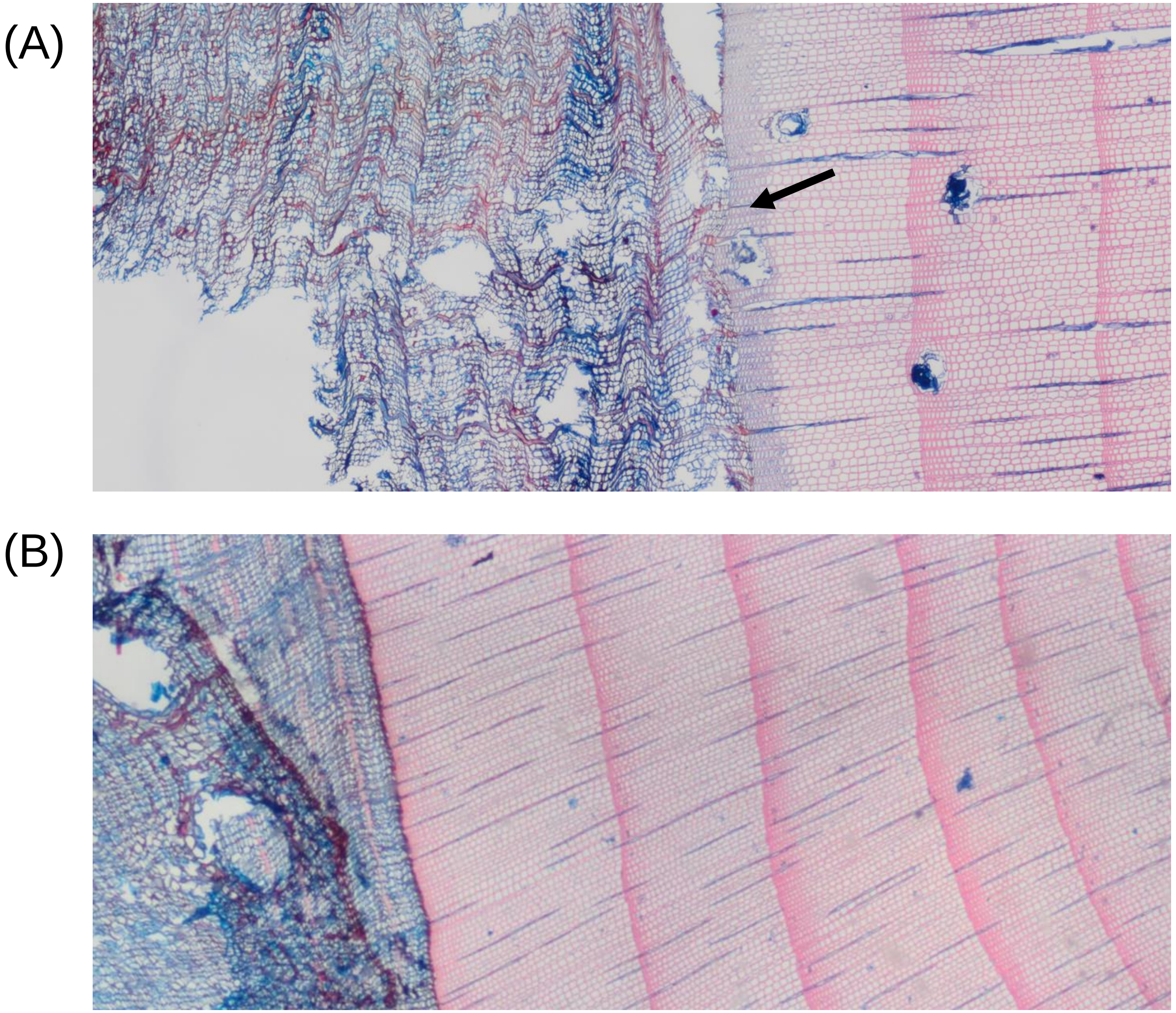

SUPPLEMENTARY FIGURE 4. Example of cambial activity in A) *Pinus sylvestris* tree and B) *Juniperus spp.* shrub from Iškoras Mountain (NE Norway) at the time of samples collection (August 11, 2022). Black arrow indicates latewood formation in the *Pinus* tree

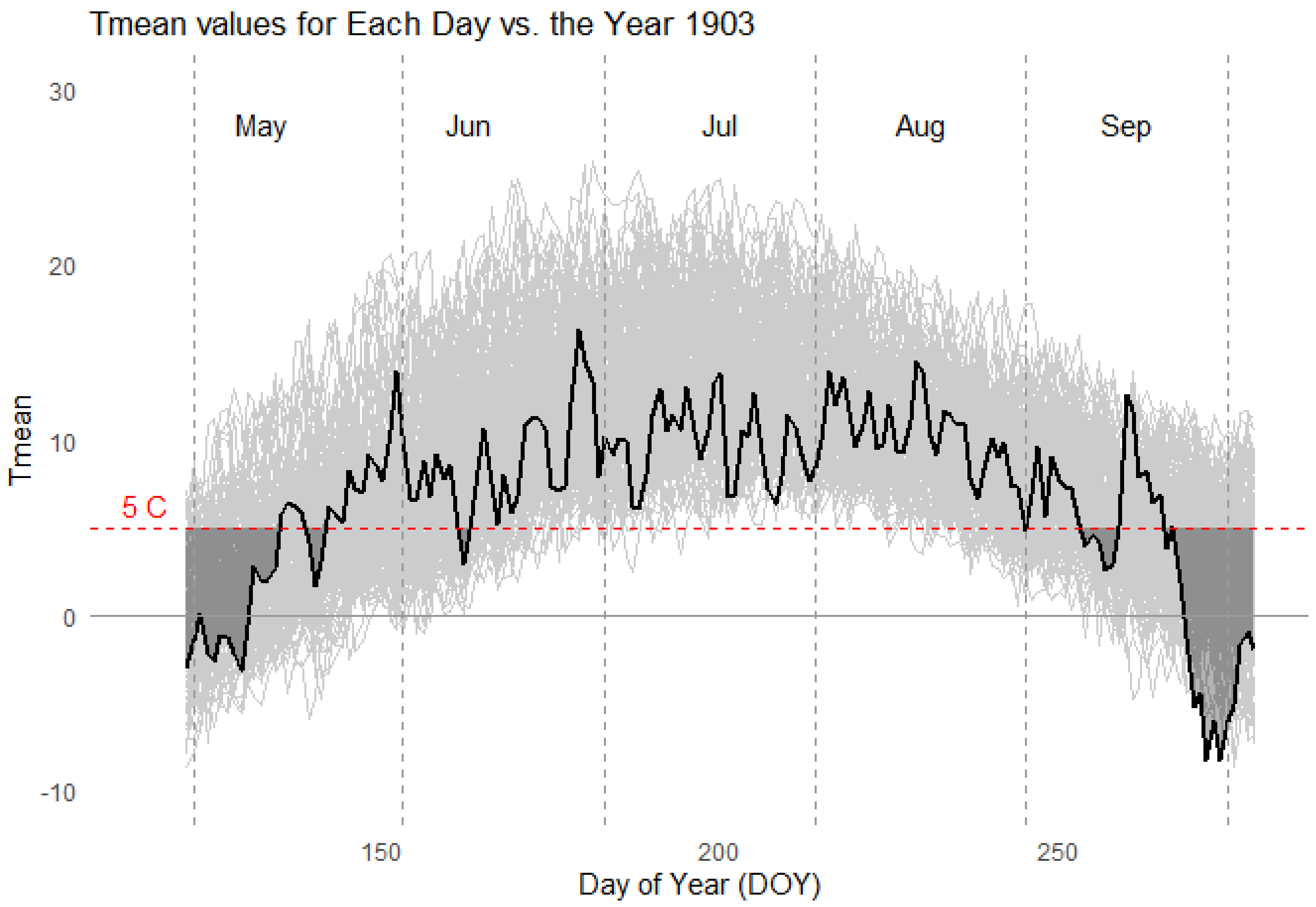

SUPPLEMENTARY FIGURE 5. Daily temperature data for the instrumental period (1877-2022, grey lines) with temperature conditions shown for year 1903 (black line) following the 1902 blue ring. Days with daily temperature < 5°C are shaded in dark grey. Data source: https://seklima.met.no/

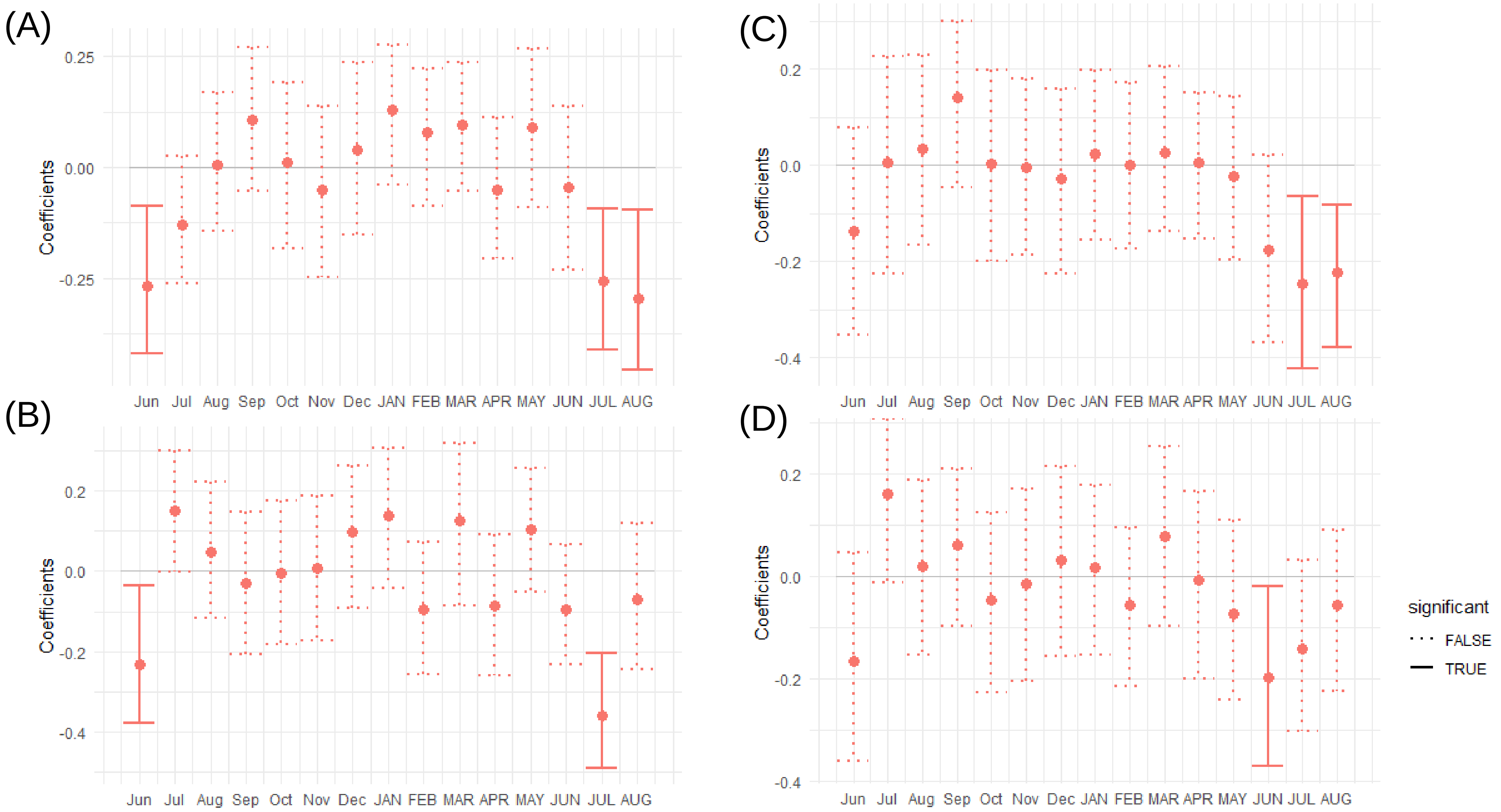

SUPPLEMENTARY FIGURE 6. Bootstrapped correlation coefficients between residua (A, B) *Pinus sylvestris* trees and (C, D) *Juniperus spp.* shrubs chronologies from Mount Iškoras (NE Norway) and (Standardized Precipitation Evaporation Index (SPEI) calculated for time steps of (A, C) three months (scale = 3) and (C, D) one month (scale = 1). SPEI data were acquired from Climate Explorer for period (1901-2018). Significant coefficients ($P < 0.05$) together with associated confidence intervals (at the level of 95%) are marked with solid lines. Monthly climatic variables include the period from the previous year's June until the current August (capital letters).
